# Primary malignant melanoma arising from ruptured ovarian mature cystic teratoma with elevated serum CA 19–9: a case report and review of literature

**DOI:** 10.1186/s12905-019-0853-8

**Published:** 2019-11-27

**Authors:** Won Ku Choi, Dong Hyun Lee, Dong Hyu Cho, Kyu Yun Jang, Kyoung Min Kim

**Affiliations:** 1 Department of Obstetrics and Gynecology, Chonbuk National University Medical School, Research Institute of Clinical Medicine of Chonbuk National University-Biomedical Research Institute of Chonbuk National University Hospital, Jeonju, Republic of Korea; 2 Department of Pathology, Chonbuk National University Medical School, Research Institute of Clinical Medicine of Chonbuk National University-Biomedical Research Institute of Chonbuk National University Hospital, 567 Baekje-daero, Dukjin-gu, Jeonju, 54896 Republic of Korea; 3 Research Institute for Endocrine Sciences, Chonbuk National University Medical School, Jeonju, Republic of Korea

**Keywords:** Ovary, Mature cystic teratoma, Melanoma, Primary

## Abstract

**Background:**

Ovarian mature cystic teratomas comprise tissues derived from all three germ layers. In rare incidences, malignant tumors may arise from ovarian mature cystic teratoma, which occurs in 0.2–1.8% of cases. A variety of tumors can arise within mature cystic teratoma, among which malignant melanoma is exceedingly rare.

**Case presentation:**

A 42-year-old woman presented with abdominal pain. Transvaginal ultrasonography showed mixed echogenic cystic masses in both ovaries. Her serum cancer antigen (CA19–9) level was elevated at 29,770 U/ml. Surgical excision was performed. Histologic examination showed infiltrating nests of pleomorphic cells with prominent nucleoli and black pigments in the background of a mature cystic teratoma. These pleomorphic cells showed strong immunoreactivity for Melan-A and HMB-45. The patient was re-evaluated and the possibility of a melanoma at any other site was ruled out. Based on these findings, we concluded that the malignant melanoma originated from the ovarian mature cystic teratoma.

**Conclusion:**

We report a rare case of primary malignant melanoma derived from an ovarian mature cystic teratoma.

## Background

Mature teratomas are common tumors, accounting for approximately 20% of all ovarian neoplasms [1]. They comprise mature tissues derived from two or three germ layers [1]. Malignant transformation of ovarian mature cystic teratoma is very rare, occurring in less than 2% of cases [2]. Moreover, primary malignant melanoma is extremely rare. To our knowledge, fewer than 40 cases have been published since the first report in 1901 by Andrews [3].

Diagnosis of malignant melanoma originating from ovarian mature cystic teratoma prior to operation is impossible. Furthermore, its biological behavior is not understood and effective treatment methods for such tumors have not been suggested due to its rarity.

Herein, we report our experience with a case of primary malignant melanoma derived from a ruptured ovarian mature cystic teratoma and associated chemical peritonitis in a 42-year-old woman.

## Case presentation

**Fig. 1:**
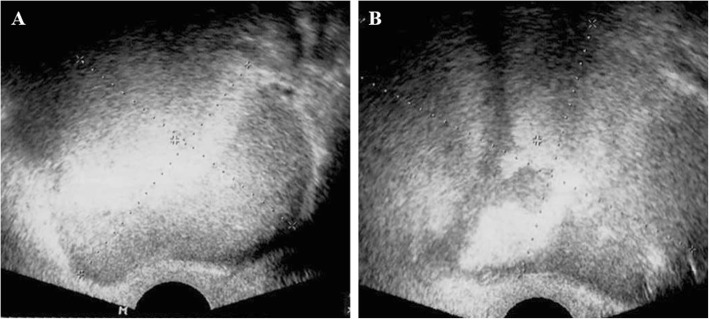
Transvaginal ultrasonography showing mixed echogenic masses in both right (**a**) and left (**b**) adnexa

A 42-year-old woman presented to the emergency department with diffuse abdominal pain and distension for the previous 5 days. Physical examination revealed a distended abdomen with marked tenderness and rebound tenderness in her lower abdomen. She had a fever at 39 °C, a pulse rate of 80/min, and blood pressure of 140/80 mmHg. Laboratory investigations showed that WBC count of 17,320 cells/mm3, hematocrit 32.6%, platelets 263,000 cells/mm3, ESR 150 mm/hr., and CRP 88 mg/L. Additionally, serum CA19–9 was elevated to 29,770 U/ml. Transvaginal ultrasonography showed cystic masses in both adnexa with mixed echogenicity and maximum diameter up to 9 cm (Fig. 1). Considering the clinical symptoms and ultrasound findings, we suspected chemical peritonitis due to a ruptured ovarian cystic mass. Under general anesthesia, we performed emergency exploratory laparotomy for confirmative diagnosis and treatment.

The surgical findings revealed approximately 1000 ml of ascites including hair and sebaceous material. Multiple dense adhesions were present between the omentum and bowel loops. The ovaries were fiable and bled easily, and the dermoid cystic material was noted in the cyst beds. Additionally, brownish black colored solid mass was identified within the cyst. We proceeded with cyst enucleation, partial omentectomy, and removal of all visible dermoid material in the abdominal cavity.

**Fig. 2:**
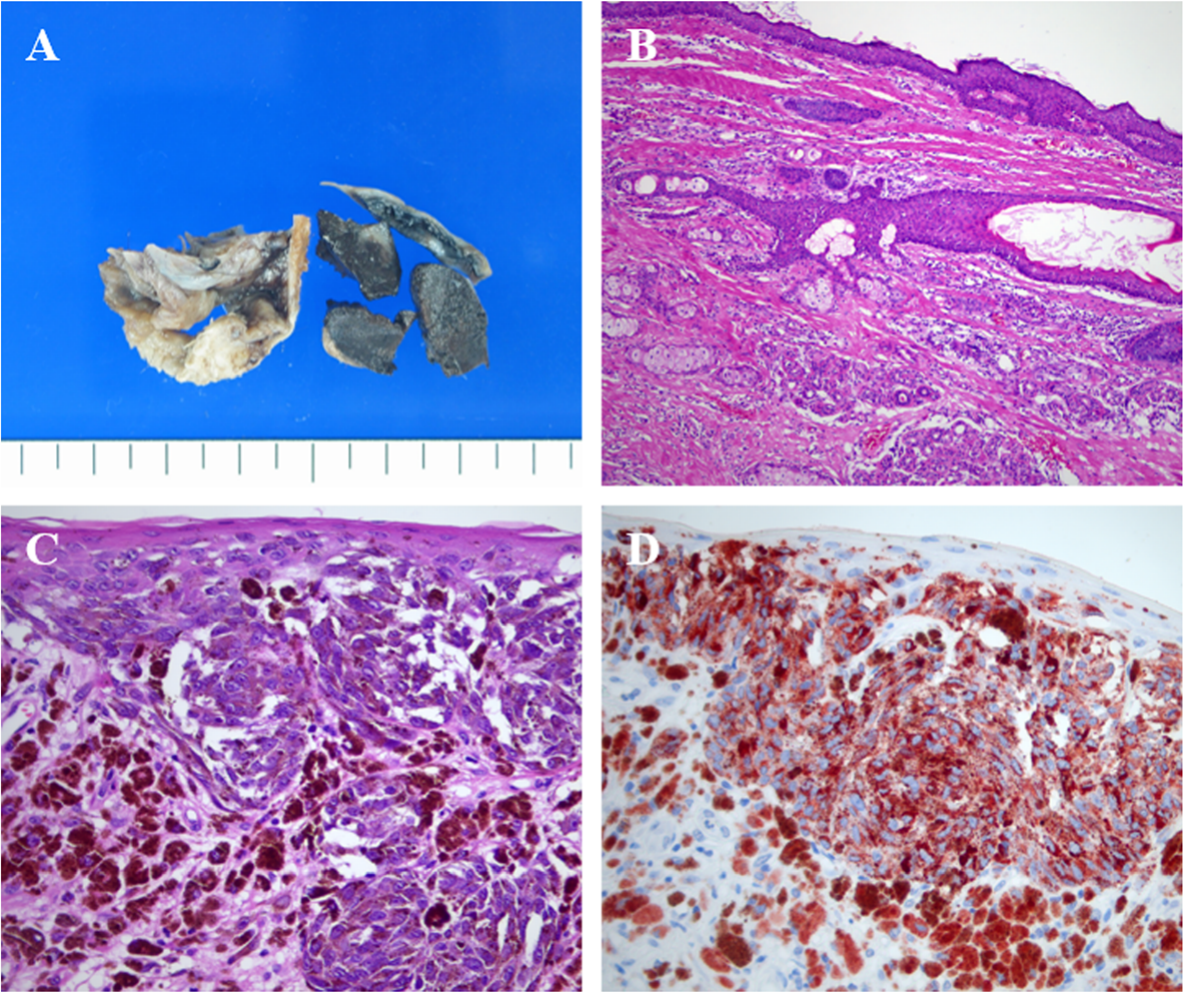
Gross and histologic features of the ovarian cystic mass. **a** Dermoid components including hairs and sebaceous materials are evident and a brownish-black colored solid mass is also present within the cyst. **b** Most of the cystic mass comprises mature dermoid components (H&E stain, original magnification × 100). **c** The solid mass contains infiltrating nests of pleomorphic cells with prominent nucleoli and black pigments. Junctional activity is also present (H&E stain, original magnification × 400). **d** The pleomorphic cells show strong immunoreactivity to HMB-45 (original magnification × 400)

Histologic examination showed that most of the cystic mass was composed of mature dermoid components (Fig. 2b). However, the brownish-black colored solid mass was composed of infiltrating nests of pleomorphic cells with prominent nucleoli and black pigments (Fig. 2c). These pleomorphic cells showed strong immunoreactivity for melan-A and HMB-45 (Fig. 2d). Based on these findings, the patient was diagnosed with malignant melanoma. The subsequent staging operation included total abdominal hysterectomy, both adnexectomy, omentectomy, appendectomy, peritoneal biopsy, and bilateral pelvic lymphadenectomy. After the surgery, the patient’s entire body was evaluated to exclude the possibility of a malignant melanoma at any other site. We finally concluded that the malignant melanoma originated from the mature cystic teratoma of the ovary. The patient remains alive and without recurrence 4 years after treatment.

## Discussion and conclusion

Although germ cell-derived ovarian mature cystic teratomas comprise 10–20% of ovarian tumors, their development to malignant tumors is rarely reported [1, 2]. Various types of tissue derived from three types of germ layer make up ovarian mature cystic teratomas and the malignant tumor that may develop from these teratomas includes squamous cell carcinoma, adenocarcinoma, sarcoma, malignant melanoma, etc. [2].

Malignant tumors secondarily arising from ovarian mature teratomas can occur at any age. However, the occurrence is most common in menopausal women ranging in age between 50 and 60 years [4]. Similar to general ovarian tumors, including lower abdominal pain, discomfort, abdominal palpable mass, dysuria, and dyschezia occur due to ovary enlargement.

Primary malignant melanomas of the female genital tract most commonly occur in the vagina, followed by the vulva [5]. Primary malignant melanomas of the ovary are exceedingly rare. Since there are no melanocytes in normal ovaries, primary malignant melanomas are thought to derive from mature teratomas [5]. Although several reports of primary ovarian malignant melanoma without evidence of mature teratoma have been published, those tumors were assumed to derive from mature teratomas and subsequently destroyed by the malignant melanoma [6].

The histologic features of ovarian malignant melanomas are similar to those of melanomas originating from the skin [5]. The commonly observed cytomorphologic features of the tumor cells include discohesion, prominent nucleoli, cytoplasmic vacuolization, and multinucleation [5]. The tumor cells are mostly arranged in diffuse solid patterns, although nested, pseudopapillary, or follicular patterns are also reported [7]. For definite diagnosis, melanocytic markers such as S-100, Melan-A, and HMB45 antibodies are commonly used for immunohistochemistry [5]. Histopathologic features of our present case were all consistent with above mentioned findings (Fig. 2).

Primary malignant melanomas of the ovaries should be distinguished from metastatic ovarian melanoma since the majority of ovarian malignant melanomas developed via metastasis from tumors in other regions [8]. The reported duration between the primary malignant melanoma of the skin and the metastatic tumor of the ovary ranges from 15 to 228 months [8]. Therefore, the primary cutaneous tumor may be quite remote from the metastatic tumor and we should carefully evaluate the history of primary cutaneous lesions before diagnosing primary malignant melanoma of the ovary.

The studies performed by Boughton et al. [9] and Cronje and Woodruff [10] used the following criteria for the diagnosis of primary malignant melanoma arising from ovarian mature teratoma: 1) No evidence of other possible primary tumor sites. 2) Malignant melanoma present in unilateral ovarian teratoma. 3) Good correlation between patient age and symptoms with those of well-documented cases in the literature; that is, middle-aged to elderly, and pain and swelling lasting several months. 4) Well-demonstrated junctional activity. However, this junctional activity is not a necessary finding for diagnosis as it was present in only 50% of reported cases. Fortunately, we were able to identify junctional activity in our present case.

Rupture of ovarian mature cystic teratoma is rare, occurring in 1.2–3.8% of cases; the low rate is attributed to the relatively thickened capsule [7]. Despite its low incidence, the rupture of ovarian mature cystic teratoma within the abdominal cavity causes granulomatous peritonitis due to acute peritonitis or continuous leak [7]. For ovarian teratoma, both direct rupture into the abdominal cavity and indirect rupture into the abdominal cavity-adjacent-luminal organs including the vagina, bladder, intestine, and rectum have been reported [7]. In case of indirect rupture into adjacent organs, diagnosis before surgery could be relatively simple based on the presence of oily secretions mixed with hair from the vagina, urethra, and rectum [7]. In contrast, rupture of the tumor into the abdominal cavity is often difficult to diagnose [7]. These patients usually complain of mild and non-specific lower abdominal pain and a sense of abdominal distension [7].

Increased serum CA19–9 levels have been proposed as a diagnostic tool for ovarian mature cystic teratoma [11]. CA19–9 is a sialylated Lewis A antigen associated with mucins in gastrointestinal adenocarcinomas and ovary mucinous carcinomas [11]. Since ovarian mature cystic teratomas are composed of cells derived from every stratum germinativum, serum CA19–9 levels are generally elevated in these patients. In the present case, an extreme increase in serum CA19–9 level was observed prior to surgery, which decreased after tumor removal, consistent with the previous reports.

Although the biological behavior of ovarian malignant melanoma is rarely evaluated and no consistent suggestions on effective treatment are available due to its scarcity, older patients who do not intend to become pregnant may undergo total abdominal hysterectomy and bilateral salpino-oophorectomy followed by omentectomy when required [12]. In contrast, younger patients who need to preserve their fertility, the adnexa of the opposite side may be conserved with careful follow-up observation for relapses [12]. No definite report has indicated improvement of prognosis by additional therapies including chemotherapy and radiotherapy after surgical treatment. However, dacarbazine (DTIC) monotherapy or combination therapy including nitrosourea and cisplatin may be administered as adjuvant therapies for relapsing or persistent lesions [12]. Recently, the positive effect of interferon in patients with melanoma with high risks of relapse was reported [13]. Although immunotherapy using recombinant cytokines, vaccines, and monoclonal antibodies has been tried, it was used only as adjuvant therapy for primary treatment; therefore, more definitive verification is required.

Primary ovarian malignant melanomas show a worse prognosis than those for ovarian carcinoma of similar stages [5]. In one report, 53% of patients died due to progressive disease with distant metastasis to other organs such as the lymph nodes, liver, and bone [12]. Patients with ascites, cyst rupture, adhesions, full-thickness cyst wall invasion, and macroscopic dissemination tend to show poor prognosis [14]. In cases of primary ovarian melanoma, the Clark/Breslow staging system for dermoid-associated melanoma has been reported to better estimate the prognosis compared to Federation of Obstetrics and Gynecology staging [15]. In this regard, patients with junctional activity had lower rates of distant metastasis and higher survival rates compared to those in patients without junctional activity [5].

In conclusion, we experienced an extremely rare case of primary malignant melanoma arising from an ovarian mature cystic teratoma in a 42-year-old woman and have reported this case and literature review. Awareness that malignant melanomas also can originate from ovarian mature cystic teratomas is important to avoid misdiagnosis.

## Data Availability

All data generated or analyzed during this study are included in this published article.

## Abbreviations

CRP: C-reactive protein
ESR: Erythrocyte sedimentation rate
